# An Electronic Nose as a Non-Destructive Analytical Tool to Identify the Geographical Origin of Portuguese Olive Oils from Two Adjacent Regions

**DOI:** 10.3390/s22249651

**Published:** 2022-12-09

**Authors:** Nuno Rodrigues, Nuno Ferreiro, Ana C. A. Veloso, José A. Pereira, António M. Peres

**Affiliations:** 1 Centro de Investigação de Montanha (CIMO), Instituto Politécnico de Bragança, Campus de Santa Apolónia, 5300-253 Bragança, Portugal; 2 Laboratório Associado para a Sustentabilidade e Tecnologia em Região de Montanha (SusTEC), Instituto Politécnico de Bragança, Campus de Santa Apolónia, 5300-253 Bragança, Portugal; 3 Instituto Politécnico de Coimbra, ISEC, DEQB, Rua Pedro Nunes, Quinta da Nora, 3030-199 Coimbra, Portugal; 4 CEB—Centre of Biological Engineering, University of Minho, Campus de Gualtar, 4710-057 Braga, Portugal; 5 LABBELS—Associate Laboratory, Braga/Guimarães, Portugal

**Keywords:** EVOO quality, sensory analysis, oxidative stability, metal oxide semiconductor sensors, multivariate qualitative-quantitative analysis, resistance electrical signals, feature extraction parameters

## Abstract

The geographical traceability of extra virgin olive oils (EVOO) is of paramount importance for oil chain actors and consumers. Oils produced in two adjacent Portuguese regions, Côa (36 oils) and Douro (31 oils), were evaluated and fulfilled the European legal thresholds for EVOO categorization. Compared to the Douro region, oils from Côa had higher total phenol contents (505 versus 279 mg GAE/kg) and greater oxidative stabilities (17.5 versus 10.6 h). The majority of Côa oils were fruity-green, bitter, and pungent oils. Conversely, Douro oils exhibited a more intense fruity-ripe and sweet sensation. Accordingly, different volatiles were detected, belonging to eight chemical families, from which aldehydes were the most abundant. Additionally, all oils were evaluated using a lab-made electronic nose, with metal oxide semiconductor sensors. The electrical fingerprints, together with principal component analysis, enabled the unsupervised recognition of the oils’ geographical origin, and their successful supervised linear discrimination (sensitivity of 98.5% and specificity of 98.4%; internal validation). The E-nose also quantified the contents of the two main volatile chemical classes (alcohols and aldehydes) and of the total volatiles content, for the studied olive oils split by geographical origin, using multivariate linear regression models (0.981 ≤ *R*^2^ ≤ 0.998 and 0.40 ≤ RMSE ≤ 2.79 mg/kg oil; internal validation). The E-nose-MOS was shown to be a fast, green, non-invasive and cost-effective tool for authenticating the geographical origin of the studied olive oils and to estimate the contents of the most abundant chemical classes of volatiles.

## 1. Introduction

Olive oil is highly appreciated worldwide due to its unique and distinct flavor, recognized nutritional properties, and beneficial health effects. Due to its price and increasing demand by consumers, extra virgin olive oil (EVOO) is one of the most prone to mislabeling [1]. Several fraud practices have been detected in the olive oil market, including the mislabeling of the oil category level or origin (cultivar, region, and production mode), and the deliberated incorporation of other edible oils. In this sense, recent studies have shown that consumers, producers, retailers, importers, exporters, as well as the regulatory bodies, understand the importance of oil traceability, namely, geographical traceability [1,2,3]. In the few last years, targeted and non-targeted strategies have been developed, aiming to address this important commercial and legal challenge, i.e., the authentication of olive oil geographical origin. Data generated by different analytical techniques (e.g., chromatography, spectrophotometry, spectroscopy, and electrochemistry) in combination with unsupervised or supervised chemometrics tools, have been proposed. For example, Hmida et al. [4] used fatty acids and triacyglycerids as origin markers of virgin olive oil (VOO) from the Mediterranean region (e.g., Portugal, France, Tunisia, and Turkey), by applying principal component analysis (PCA). Furthermore, PCA of the fatty acids and volatiles profiles of Tunisian olive oils identified the oil’s geographical origin [5]. The composition of volatile compounds was also successfully used as geographical origin markers of Greek olive oils of cv. ‘Ntopia’ [6]. Quintanilla-Casas et al. [7] proposed using the sesquiterpene hydrocarbon fingerprint together with partial least squares-discriminant analysis (PLS-DA) to verify EU and single-country origin label declaration. Recently, the use of inorganic multi-elemental and isotopic signatures in olive oils as possible geographical traceability strategies was reviewed, highlighting the possible use of trace elements as origin markers [8]. Alternatively, nuclear magnetic resonance spectroscopy, coupled with different multivariate statistical tools, has been revealed as a potential tool for identifying the geographical origin of olive oils [9]. However, these strategies are destructive, requiring several pre-treatments of the olive oil samples; non-green, relying in the use of several organic solvents; expensive, using non-affordable apparatus; and, time-consuming. On the other hand, the research team has recently demonstrated the possibility of using Fourier transform infrared spectroscopy (FTIR), coupled with linear discriminant analysis (LDA), as a green, fast, and non-destructive approach to identify the geographical origin of Portuguese olive oils from cv. ‘Galega Vulgar’ [10]. FTIR coupled with the machine learning technique was also previously used by Scatigno and Festa [11] to identify spectroscopy benchmarks in Italian EVOO, allowing for the recognition of the oils’ geographical origin. These researchers also verified the successful geo-discrimination of Italian EVOO based on energy dispersive X-ray fluorescence data acquired using a portable spectrometer that perform in situ, fast, and non-destructive elemental and molecular analysis, combined with different chemometric tools (e.g., PC; soft independent modeling of class analogy, SIMCA; or, L-shaped PLS Regression, L-PLSR) [12]. Fluorescence and FT-Raman spectroscopy coupled with PCA or PLS–DA were also applied to authenticate the geographical origin of Arbequina EVOOs from two geographically adjacent Spanish regions [13].

In this study, and to the authors’ best knowledge, it was intended to evaluate, for the first time, the potential application of a lab-made electronic nose (E-nose) with metal oxide semiconductor (MOS) sensors as a tool for identifying the geographical origin of olive oils produced in two adjacent Portuguese regions (northeast of Portugal), from Côa and Douro. E-noses provide an olfactory unique fingerprint of the olive oil’s volatiles, which, together with multivariate statistical techniques, leads to their successful use in the olive oil industry, namely as a classification sensor-aroma tool [14,15,16], to detect EVOO adulteration with other oils [17,18], or to check the olive oil quality grade category [19].

## 2. Materials and Methods

### 2.1. Olive Oil Samples

**Figure 1:**
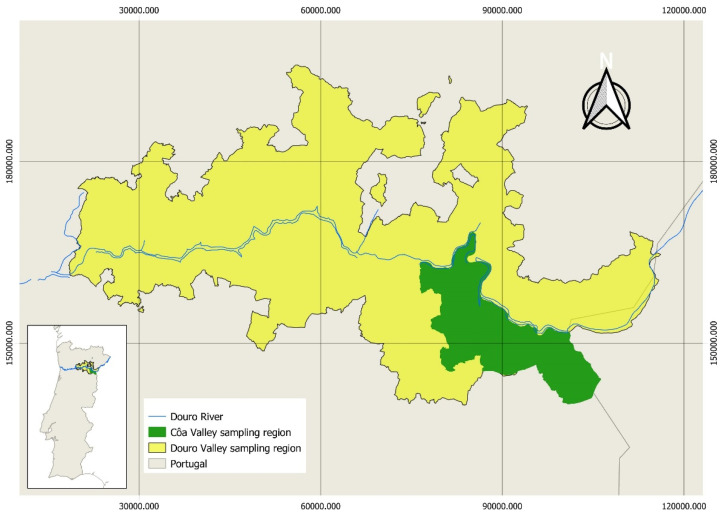
Côa Valley and Douro Valley sampling regions. Map projected in ETRS89/PT-TM06.

The olive oils were directly collected from olive mills located in the regions of Côa and Douro (Figure 1), northeast of Portugal, during 2021. In each olive mill, two olive oil bottles (500 mL) per lot were collected. The amber glass bottles were transported to the laboratory and stored in a dark environment at room temperature (18–25 °C), and were only filtered before analysis. In total, 31 independent samples were collected in the Douro Valley and 36 in the Côa Valley. Olive oils were extracted in 21 different olive oil mills, of which 13 were located in the Douro Valley region and 8 were located in the Côa Valley region. According to the producers, all samples were extracted from traditional Portuguese olive cultivars; olives with maturation indices ranging from 2.5 to 5. In the Douro Valley, the predominant olive varieties are cvs. Galega, Madural, Cordovil, and Cobrançosa. In Côa Valley, the predominant varieties are cvs. Madural, Negrinha de Freixo, Verdeal Transmontana, and Redondal. In general, no monovarietal olive oils are produced in these regions; the majority of the olive oils are blends of different varieties.

### 2.2. Quality Physicochemical Parameters, Oxidative Stability, Total Phenols Content, and Sensory Analysis

Free acidity (FA, % oleic acid), peroxide value (PV, mEq O_2_/kg oil), and specific extinction coefficients at 232 nm and 268 nm (*K*_232_ and *K*_268_) of the olive oils were determined using the regulated European Union (EU) standard methodologies [20].

The oxidative stability (OS, h), an indication of the oil’s shelf-life, was determined by monitoring the oil’s oxidation induction time, as described in the literature [21], using Rancimat 743 equipment (Metrohm CH, Switzerland).

The total phenols content (TPC) was determined using the Folin-Ciocalteu method, by recording the absorbance at 765 nm of a methanol-water extract of each olive oil in a UV-VIS/UV-1280 Shimadzu spectrophotometer (Shimadzu Europa GmbH, Duisburg, Germany). The results were expressed in Gallic acid equivalent (mg GAE kg^−1^), using a previously established calibration curve [15,22].

The antioxidant capacity of the olive oil samples was spectrophotometrically assessed regarding the radical scavenging activity of DPPH (2,2-diphenyl-1-picrylhydrazyl) (DPPH). The assays were conducted in a spectrophotometer (UV–VIS/UV-1280 Shimadzu), at 20 °C and 517 nm, following the methodology described by Cherif et al. [23] with some modifications. The solution of DPPH used as the control and olive oil extracts were prepared by mixing 0.5 mL of methanol and 3.5 mL of DPPH (0.06 mM), and the absorbance values were read after 30 min in the dark. The DPPH radical scavenging was expressed as the reduction percentage of the DPPH activity.

The olfactory and gustatory-retronasal profiles of the olive oils were established by a trained sensory panel of the Polytechnic Institute of Bragança (Bragança, Portugal), following the methodology of the EU regulation [20] and the International Olive Council (IOC) [24], using a modified continuous intensity scale varying from 0 (no perceived sensation) to 10 (maximum perceived intensity), as previously discussed [21]. The sensory panel included 8 trained members as previously described [25], with the following sensations graded: fruitiness (ripe or green), fruit sensations, herbal sensations, as well as the sweetness, bitterness, and pungency sensations. Moreover, the harmony, complexity, and persistence were also evaluated.

### 2.3. Chromatographic Profile of the Volatile Fraction of the Studied Olive Oils

Olive oil volatiles were assessed by headspace solid phase microextraction (HS-SPME) and gas chromatography (GC-2010 Plus; Shimadzu) with a mass spectrometer (MS) detector (GC/MS-QP2010 SE; Shimadzu) following the methodology described by Silva et al. [26]. Briefly, in a 50 mL glass vial, 3 g of olive oil were mixed with 5 µL of a 0.125 mg/mL solution of 4-methyl-2-pentanol (Sigma-Aldrich, St. Louis, MO, USA), which was used as the internal standard. To enhance the release of volatiles, the vial was heated at 40 °C and agitated at 300 rpm for 5 min, which were then adsorbed for 30 min by a SPME fiber (divinylbenzene/carbonex/polydimethylsiloxane 50/30 µm; Supelco, Bellefonte, PA, USA). Afterwards, thermal desorption of the adsorbed volatiles was accomplished at the injection port of the chromatograph (220 °C, 1 min), and the fiber was cleaned and conditioned before the next assay. Peaks separation was performed in a TRB-5MS column (30 m × 0.25 mm × 0.25 μm; Teknokroma, Spain), using helium (Praxair, Portugal) as the mobile phase (30 cm/s, 24.4 mL/min) for the samples manually injected (splitless mode). During each run, a temperature gradient was applied (40 °C for 1 min followed by an increase of 2 °C/min to 220 °C during 30 min). The temperature of the ionization source was set equal to 250 °C, with a related energy of 70 eV, and a current of 0.1 kV. All mass spectra were acquired by electronic ionization in the range of 35–500 m/z. Compounds were identified considering the mass spectra and the Kovat’s indices (NIST SRD-69 Library from National Institute of Standards and Technology, Gaithersburg, MD, USA; and, the free chemical structure/information databases of PubChem and ChemSpider). The area of each peak was assessed by integration of the total ion chromatogram. The semi-quantitation of the volatile compounds was based on the relative area of each peak, being transformed into a mass equivalent using the known mass of the added internal standard. Each sample was evaluated in triplicate.

### 2.4. E-Nose Analysis

#### 2.4.1. Lab-Made Device

The characteristics of the lab-made E-nose have been previously described in the literature [14,15,27]. Before analysis, the oil samples were heated at 28 °C; detection at the sensor’s unit was performed in a controlled heated environment (35 °C). For cleaning purposes, as well as to ensure the fast delivery of the vapor headspace of each sample to the sensors’ chamber, a diaphragm vacuum air pump (model SC3502PM, from SKOOCOM, Shenzhen, Guangdong, China) was used. For cleaning purposes (system and sensors), atmospheric air was pumped at a constant flow until a stabilized baseline was observed for all the E-nose sensors. Nine commercial MOS gas sensors were included in the device (S1: TGS 2600 B00; S2: TGS 2602; S3: TGS 2610 C00; S4: TGS 2611 C00; S5: TGS 2610 D00; S6: TGS 2611 E00; S7: TGS 2612; S8: TGS 826 A00; and S9: TGS 823 C12N). The adsorption of the volatile compounds released by each olive oil into the surface of the TGS sensors led to changes of the electrical properties; the electrical resistances (in ohms, Ω) were recorded by an Agilent data acquisition unit (model 34970A) and monitored using Agilent BenchLink Data Logger software.

#### 2.4.2. Olive Oil Analysis and Signal Processing

As previously described [14,15], for the analysis, 0.5 mL of oil were placed in a glass vial (25 mL) that was inserted in the sampling chamber (13 min at 28 °C). Simultaneously, the E-nose device was cleaned using atmospheric air until a stable signal baseline was recorded for all nine TGS sensors. Then, the gas headspace contained in the vial was pumped into the detection chamber, where it interacted with the sensors for 2.5 min; the electrical resistance was recorded every 4 s.

The recorded electrical resistances by each of the nine TGS sensors were processed using seven feature extraction methods [28]: the last response point (LP), the integral of the response curve (INT), the maximum response point (MAX), the minimum response point (MIN), the sum of the response curve (SUM), the mean of the response curve (MEAN), and the standard deviation of the signal responses (SD).

### 2.5. Statistical Analysis

Statistical differences of oil composition/characteristics related to the two adjacent geographical origins under study (Côa versus Douro) were evaluated using the *t*-Student test without or with the Welch’s correction, depending on if equal or unequal variances between groups could be assumed or not, respectively. This latter requirement was assessed by applying the F-test to two sample variances. PCA was applied as an unsupervised pattern recognition tool to infer the possible use of the physicochemical parameters (67 olive oils × 7 parameters), sensory data (67 olive oils × 10 olfactory sensations or 67 olive oils × 15 gustatory sensations), volatiles profiles (67 olive oil × 8 volatiles chemical classes), and E-nose olfactory fingerprints (for each of the seven feature extraction technique: 67 olive oils × 9 signal responses) to differentiate between the studied olive oils according to their geographical origin. LDA coupled with the simulated annealing (SA) algorithm was applied to identify the most powerful non-redundant discrimination E-nose sensors, based on the best correct classification performances (i.e., sensitivities and specificities) obtained for the leave-one-out cross-validation (LOO-CV) procedure. Multiple linear regression models (MLRM) were also established based on selected responses from each dataset regarding the seven feature extraction techniques used, in order to estimate the volatiles content of the most abundant chemical classes, as well as of the total volatiles content. The quantitative performance was discussed based on the determination coefficients (*R*^2^) and on the root mean square errors (RMSE). Furthermore, the possible use of the developed models as an alternative analytical procedure to the chromatographic standard method, usually applied for volatile’s content determination, was further evaluated according to the XPT 90-210 French standard [29]. The statistical analysis was performed using the open-source packages of the R statistical program (RStudio version 2021.09.0; “Ghost Orchid” Release (077589bc, 20 September 2021)), at a 5% significance level.

## 3. Results and Discussion

### 3.1. Quality Parametes, Oxidative Stability, Antioxidant Capacity, Sensory, and Volatiles Profiles of Olive Oils from Côa and Douro Adjacent Geographical Regions

The 67 independent olive oils from the two geographically adjacent regions were evaluated, considering the physicochemical quality parameters used for assessing the quality grade category (i.e., FA, PV, *K*_232_ and *K*_268_). The OS, the TPC, and DPPH capacity were determined for each olive oil. Table 1 shows the mean values of each parameter concerning the olive oils studied for each region (Côa: 36 oils; and, Douro: 31 oils). As can be inferred from the statistical analysis, with the exception of the FA, the other evaluated parameters were significantly influenced (*p*-value < 0.05) by the oils’ geographical origin, even if the regions were adjacent. Olive oils from the Côa region showed higher PV and extinction coefficient values compared to those from Douro, but they also showed significantly greater OS, TPC, and DPPH activity (*p*-value < 0.05). It should be noted that, independent of geographical origin, all olive oils fulfilled the legal thresholds established by the European Community Regulation EEC/2568/91 of 11 July, and subsequent amendments for the classification as Extra Virgin Olive Oil (EVOO) category (FA ≤ 0.8 % oleic acid, PV ≤ 20 mEq O_2_ kg^−1^, *K*_232_ ≤ 2.5 and, *K*_268_ ≤ 0.22, respectively).

The influence of the geographical origin on the sensory profiles of the olive oils was also studied. Table 2 reports the mean intensities of the olfactory and gustatory sensations perceived by the trained panelists, as well as, for each region, the percentage of the oils for which each specific sensation was detected (intensity > 0). The results pointed out that the geographical origin greatly affects the intensities of the olfactory-gustatory sensations of the studied oils (*p*-value < 0.05). Oils from Côa Valley could be classified as fruity-greenly oils, and, on the other hand, those from Douro Valley could be classified as fruity-ripely oils. In total, ten olfactory sensations could be perceived in the evaluated oils, although only six and four of them could be detected in the majority of the olive oils from the Côa or Douro Valleys, respectively. Similarly, fifteen gustatory sensations were perceived in the studied oils, although only eleven and eight of them were detected in more than half of the olive oils from the Côa or Douro Valleys, respectively. Furthermore, on average, oils from Côa Valley were more bitter and pungent, and those from Douro were sweeter. Overall, two main findings emerged: (i) compared to oils from both regions, in the oils from Côa Valley, the perceived olfactory and gustatory sensations were more intense (with the exception of sweet); and, (ii) in general, the percentage of oils from Côa Valley with a perceived specific olfactory or gustatory sensation was higher, pointing out that oils from Côa Valley were richer in different sensory sensations. It should be noted that several of the sensory sensations perceived (e.g., apple, banana, cabbage, dry fruits, tomato, dry herbs, fresh herbs, olive leaves, and tomato leaves) were also previously reported for olive oils produced in the Côa Valley [25] or Douro Valley [26], although at different perceived intensities, which could be tentatively attributed to the effect of different climatic conditions coupled with probable different oil extraction conditions.

**Figure 2:**
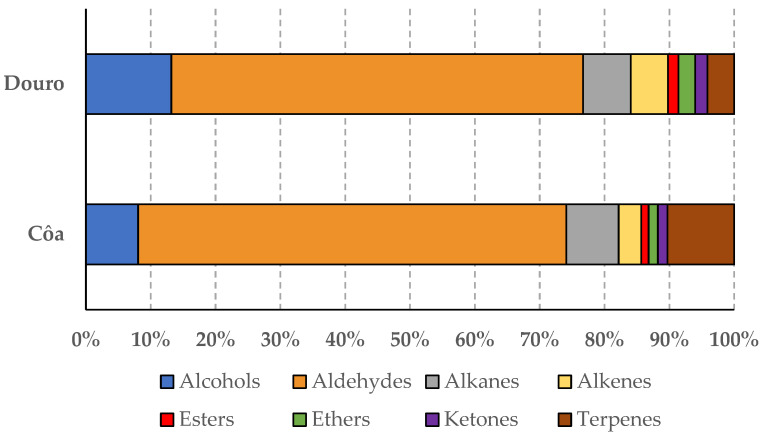
Relative abundance (%) of the chemical classes of the identified VOCs for the olive oils from Côa or Douro geographical origins.

Finally, the profiles of the volatile compounds, of the studied olive oils, were chromatographically established by HS-SPME-GC-MS. Globally, sixty-three volatiles were detected belonging to eight chemical families (i.e., fourteen alcohols, ten aldehydes, six alkanes, seven alkenes, four esters, one ether, six ketones, and fifteen terpenes). Previous studies on olive oils also produced in the Douro region detected eleven volatiles [30] and sixty-two volatiles [26], of the same chemical classes. However, it should be highlighted that in the present study, twenty-nine of the sixty-three identified volatiles were only detected in a low number of olive oils (i.e., in less of ten oils of the sixty-seven independent oils evaluated). Thus, due to this variability, which concerns the present study, it was decided to use the information gathered for each chemical class rather than the data for the individual volatile compounds. Table 3 lists the mean contents (in mg of each compound as internal standard equivalents per kg of olive oil) for each of the eight chemical classes of volatiles identified in the olive oils from each of the two regions under study. As can be seen from the results, the geographical origin significantly influenced the contents of the oils’ volatiles classes, as well as the total contents of volatiles (*p*-value < 0.05). Indeed, compared to oils from the Côa Valley, those from Douro were significantly richer in alcohols, aldehydes, alkanes, alkenes, esters, ethers, and ketones; although similar contents were found for terpenes. Moreover, for oils from both regions, aldehydes were the most abundant chemical class, representing 64–66% of the total content of volatiles, followed by alcohols (8–13%), alkanes (7–8%), and terpenes (4–10%) (Figure 2). It should be noted that although the two most abundant chemical classes were the same as those previously reported for olive oils from the Douro Valley (cvs. Carrasquinha, Cobrançosa, Cordovil, Galega, Madural, Negrinha, and/or Verdeal), the relative abundances found were quite different [26,30]. The different crop years (i.e., different climatic conditions), the different maturation indices of olives at harvest, the different olive cultivars, and respective extracted monovarietal olive oil or blends, as well as the oils’ extraction conditions, may justify the observed differences. Indeed, according to Garcia et al. [30], the volatiles content of olive oils from the Douro Valley were significantly influenced by the olive cultivar, as well as by the olives’ maturation indices at harvest. Other studies reported quite broad ranges for the relative abundances of these two VOCs’ chemical classes, namely for cv. Chaaibi olive oils produced in different Tunisian regions (0–77% and 6–86% for alcohols and aldehydes, respectively) [5] or for Italian monovarietal olive oils or blends from different cultivars (cvs. Moraiolo, Frantoio, Leccino, Borgiona, Dolce Agogia, San Felice, Raio, and/or Limona) (~5–30% and ~30–70% for alcohols and aldehydes, respectively) [31].

**Figure 3:**
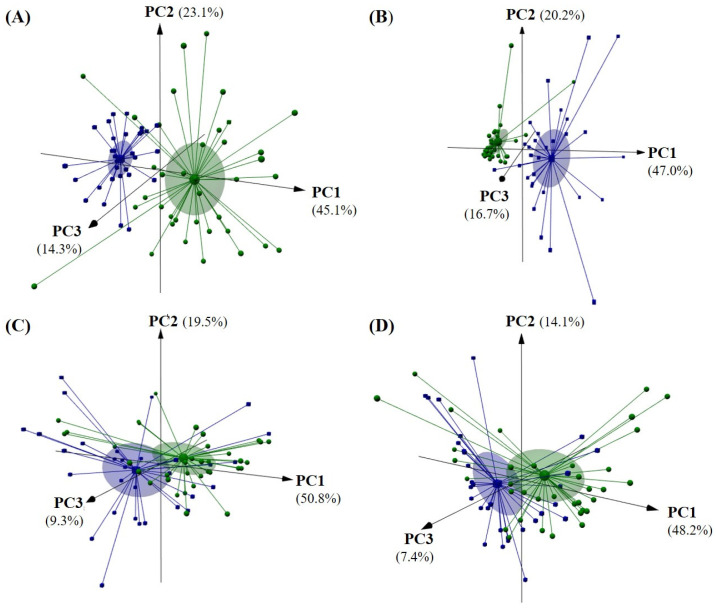
Unsupervised pattern recognition of olive oils by geographical origin (Côa Valley: ●, and, Douro Valley: ■) based on the principal component analysis (3D plot of the three first PCs) based on: (**A**) physicochemical quality data (FA, PV, *K*_232_ and *K*_268_), oxidative stability (OS), and antioxidant reducing capacity (TPC and DPPH); (**B**) contents of the volatiles belonging to eight chemical families (alcohols, aldehydes, alkanes, alkenes, esters, ethers, ketones, and terpenes); (**C**) intensities of the perceived olfactory sensations (fruity greenly or ripely, apple, banana, cabbage, dry fruits, tomato, dry herbs, fresh herbs, and tomato leaves); and (**D**) intensities of the perceived gustatory sensations (fruity greenly or ripely, bitter, sweet, pungent, apple, banana, cabbage, dry fruits, plum, tomato, dry herbs, fresh herbs, olive leaves, and tomato leaves).

The influence of geographical origin on each set of olive oil parameters was further checked by PCA. Figure 3 shows the 3D plots regarding the unsupervised differentiation of the 67 olive oils according to their geographical origin (Côa Valley versus Douro Valley). As can be seen, although significant differences were found for each set of data (Table 1, Table 2 and Table 3), quality parameters, together with stability data and antioxidant related data, in which three first PCs explained 82.5% of the data variability (Figure 3A), and the chromatographic profiles regarding the eight chemical families considered (Figure 3B), in which three first PCs explained 83.9% of the data variability, allowed the best differentiations of oils from Côa Valley (green spheres) from those of Douro Valley (blue cubes). A lower level of differentiation was achieved when using the perceived intensities of olfactory (Figure 3C) or gustatory (Figure 3D) sensations, although the oils showed unequivocally significant sensory differences (Table 2).

### 3.2. Identification of Olive Oil Geographical Origin and Assessment of the Alcohls, Aldehydes, and Total Volatiles Content of Oils from Côa and Douro Adjacent Regions

**Figure 4:**
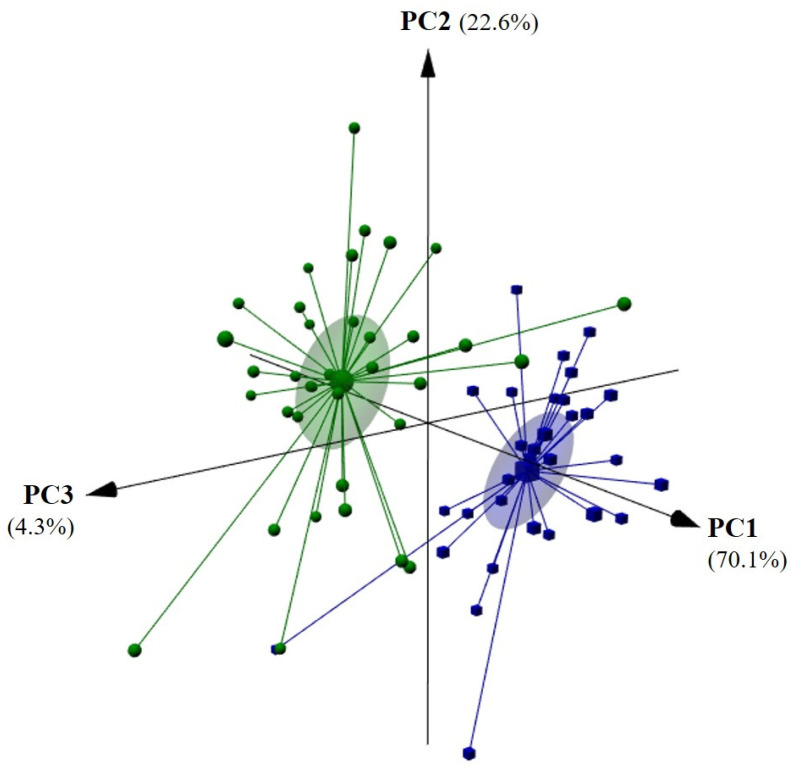
Unsupervised pattern recognition of olive oils by geographical origin (Côa Valley: ●, and, Douro Valley: ■) based on the principal component analysis (3D plot of the first three PCs) based on the mean resistance signals acquired by the nine E-nose-MOS sensors (S1_MEAN to S9_MEAN).

As previously discussed, the olive oil contents of the eight chemical classes of volatiles, and, to a less extent, the oils’ olfactory profiles, allowed for the unsupervised recognition of the oils’ geographical origin. However, gathering these data is expensive and time-consuming. Thus, an analytical approach based on the use of a self-built E-nose prototype, comprising MOS sensors, was evaluated as a non-invasive, green, cost-effective, and alternative classification tool. In fact, the variability of the volatiles emitted by each independent olive oil, in number and amount according to the region of origin (Table 3), may lead to different resistance responses by the MOS sensors of the E-nose, enhancing the application of this device for olive oil geographical origin recognition. The data gathered after applying the seven feature extraction techniques (LP, INT, MAX, MIN, SUM, MEAN, and SD) to the recorded curve of the electrical resistances with time, by each of the nine MOS sensors, were evaluated to establish unsupervised pattern recognition models. The seven data subsets, based on the feature extraction data, allowed a satisfactory unsupervised differentiation of the olive oils by geographical origin; the better split was achieved by the MEAN feature extraction approach (Figure 4). The first three PCs, which explained 97% of the data variability, allowed successful geographical origin identification.

To further evaluate the olive oil origin recognition potential of the E-nose, a LDA-SA analysis was performed. The analysis established a multivariate supervised classification model, with a single discriminant function, based on the mean resistance signals acquired by four MOS sensors (S3_MEAN, S4_MEAN, S6_MEAN, and S7_MEAN). The model successfully predicted the geographical origin of 66 of the 67 oils studied, which corresponded to an overall sensitivity of 98.5% and an overall specificity of 98.4% (one olive oil from Côa Valley was misclassified as belonging to the Douro Valley).

Lastly, since the resistance signals recorded by each MOS sensors would result from the interaction between the sensors’ membranes and the volatiles emitted by the different olive oils, the possibility of applying the E-nose to estimate the oils’ contents of alcohols and aldehydes (the two most abundant classes according to Table 3) was also studied, as well as the total content of volatiles (alcohols + aldehydes + alkanes + alkenes + ethers + esters + ketones + terpenes), released by the oils from each geographical region. Table 4 reports, for each region considered, the details regarding the MLRM established for estimating each parameter, including the number of feature extracted variables included in each model, the determination coefficients, and the root mean square errors. The results clearly showed that the MLRMs developed based on the information gathered by the E-nose-MOS device could be a promising strategy to accurately estimate the contents of the two main volatile chemical classes (alcohols and aldehydes) found in the studied olive oils, as well as of the total contents of the detected volatiles (0.981 ≤ *R*^2^ ≤ 0.998 and 0.40 ≤ RMSE ≤ 2.79 mg/kg oil).

**Figure 5:**
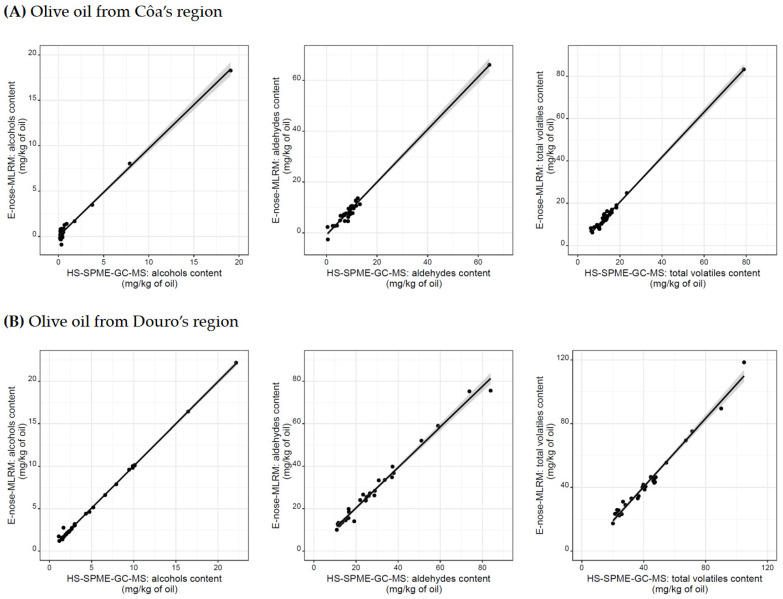
Contents (in mg/kg of oil) of volatiles predicted by the MLRMs established based on the selected feature extracted variables from the electrical resistance signal curves acquired by the nine-MOS sensors comprised on the lab-made E-nose, versus the experimental data determined by the HS-SPME-GC-MS technique.

As a final point, the application of the developed MLRMs, based on the feature extracted variables from the E-nose-MOS signal profiles, as a complementary or possible alternative technique for estimating the contents of the two volatile chemical classes and of the total content of detected volatiles in the olive oils from the Côa or Douro Valleys, was further assessed according to the XPT 90-210 French standard [29]. Therefore, for each geographical region, single linear regressions were established (Figure 5) between the volatiles content predicted by the MLRMs (Table 4) and the experimental data determined by HS-SPME-GC-MS. The quality of the fitting was examined by evaluating if the slope and intercept values were statistically equal to the theoretical values, one and zero, respectively (i.e., perfect linear fit). The results demonstrate that, at a 5% significance level, the slope and intercept values for all the developed single linear regression models were statistically equal to the expected theoretical values (the confidence intervals for the slopes included the value one, and those for the intercepts included the zero value), confirming that the E-nose-MOS could be used to accurately monitor the volatiles content of olive oils.

## 4. Conclusions

The Portuguese valleys of Côa and Douro are adjacent geographical regions, located within an UNESCO heritage site, the “Côa Valley”. In this sense, it is of utmost commercial relevance to be able to identify the geographical origin of high-value agri-food products according to each of these two regions. The present study showed that an electronic nose, comprising metal oxide semiconductors sensors, can be successfully applied as a non-invasive, fast, green, and accurate technique for recognizing the geographical origin of olive oils from the Côa or Douro Valleys. Furthermore, the lab-made device quantified the two most abundant volatiles chemical classes; i.e., the contents of alcohols and aldehydes, as well as the total volatiles content found in the studied olive oils. The satisfactory qualitative-quantitative performance of the device could be related to the observed differences found at the olfactory, as well as at the volatile, composition levels. It could be concluded that the electronic nose could be accurately used as a complementary/alternative tool to the standard techniques (e.g., chromatography). Indeed, the self-built sensor-based olfactory device showed a better differentiation-discrimination power than that achieved with the sensory profile established by trained panelists, and a similar qualitative-quantitative performance compared to that achieved by gas chromatography.

## Figures and Tables

**Table 1:** Mean value (±standard deviation) of free acidity (FA), peroxide value (PV), extinction coefficients (*K*_232_ and *K*_268_), oxidative stability (OS), total phenols content (TPC), and antioxidant activity (DPPH) of olive oils from Côa (36 independent oils) and Douro regions (31 independent oils).

Physicochemical, Stability and Antioxidant Data	Olive Oil’s Geographical Origin		*p*-Value *
	Côa Valley	Douro Velley	
FA (g_oleic acid_/100 g)	0.22 ± 0.07a	0.23 ± 0.04a	0.3085 ^#^
PV (mEq O_2_/kg)	5.63 ± 2.09a	4.82 ± 1.41b	0.0046 ^#^
*K*_232_	1.97 ± 0.29a	1.82 ± 0.27b	0.0024 ^$^
*K*_268_	0.15 ± 0.03a	0.13 ± 0.02b	<0.0001 ^#^
OS (h)	17.5 ± 7.4a	13.9 ± 6.4b	0.0017 ^$^
TPC (mg GAE/kg)	505 ± 188a	274 ± 77b	<0.0001 ^#^
DPPH (%)	53.2 ± 17.2a	13.9 ± 6.4b	<0.0001 ^#^

* Different lowercase letters correspond to statistically significant differences at a significance level of 5%. ^#^ *p*-value: *t*-Student with the Welch’s correction for unequal variances according to the *F*-test for sample variance (*p*-value < 0.05). ^$^ *p*-value: *t*-Student test when equal variances could be assumed according to the *F*-test for sample variance (*p*-value > 0.05).

**Table 2:** Intensities (in an unstructured scale from not perceived (0) to maximum intensity (10)) of olfactory, gustatory, and global sensations (mean ± standard deviation; percentage of oils with the perceived sensation) of olive oils from the Côa (36 oils) and Douro (31 oils) Valleys.

Sensory Attributes		Olive Oil Geographical Origin				*p*-Value *
		Côa Valley	Oils with Perceived Sensation	Douro Valley	Oils with Perceived Sensation	
Olfactory sensations						
Fruity	Greenly	3.4 ± 1.9a	81%	1.1 ± 1.8b	33%	<0.0001 ^$^
	Ripely	0.9 ± 2.0b	19%	2.9 ± 2.4a	67%	<0.0001 ^$^
Fruit sensations	Apple	4.5 ± 0.6a	100%	3.9 ± 1.7b	93%	0.0034 ^$^
	Banana	2.0 ± 2.4a	44%	1.9 ± 2.7a	38%	0.7603 ^$^
	Cabbage	2.5 ± 2.4a	57%	1.2 ± 2.2b	27%	0.0007 ^$^
	Dry fruits	3.4 ± 0.7a	100%	2.9 ± 1.2b	95%	0.0032 ^$^
	Tomato	4.5 ± 2.0a	89%	2.5 ± 2.4b	63%	<0.0001 ^$^
Herbaceous sensations	Dry herbs	0.9 ± 1.9b	19%	1.5 ± 2.0a	42%	0.0440 ^$^
	Fresh herbs	3.3 ± 1.9a	79%	1.2 ± 1.8b	33%	<0.0001 ^$^
	Tomato leaves	1.7 ± 2.2a	42%	1.2 ± 1.9a	30%	0.1540 ^$^
Harmony		8.0 ± 0.5a	----	7.8 ± 0.9b	----	0.0079 ^$^
Gustatory sensations						
Fruity	Greenly	1.0 ± 2.2b	19%	2.8 ± 2.6a	58%	<0.0001 ^#^
	Ripely	4.0 ± 2.3a	81%	1.6 ± 2.1b	42%	<0.0001 ^$^
Basic taste	Bitter	3.7 ± 1.3a	100%	2.3 ± 1.0b	100%	<0.0001 ^#^
	Sweet	3.4 ± 3.2b	100%	4.6 ± 2.0a	100%	0.0060 ^#^
Kinesthetic sensation	Pungent	4.4 ± 1.2a	100%	3.2 ± 1.0b	100%	<0.0001 ^$^
Fruit sensations	Apple	4.6 ± 0.8a	100%	4.2 ± 1.7b	93%	0.0462 ^#^
	Banana	2.8 ± 2.7a	56%	2.9 ± 2.8a	60%	0.7369 ^$^
	Cabbage	3.5 ± 2.7a	68%	1.1 ± 2.2b	24%	0.0429 ^#^
	Dry fruits	4.3 ± 3.6a	99%	3.2 ± 1.1	97%	0.0097 ^#^
	Plum	0.7 ± 1.6a	18%	0.3 ± 1.1b	7%	0.0370 ^#^
	Tomato	4.5 ± 1.9a	92%	2.5 ± 2.4b	56%	<0.0001 ^#^
Herbaceous sensations	Dry herbs	0.8 ± 1.9a	18%	1.4 ± 2.1a	34%	0.1291 ^$^
	Fresh herbs	3.1 ± 2.1a	75%	1.4 ± 2.0b	36%	<0.0001 ^$^
	Olive leaves	1.1 ± 2.0a	24%	0.1 ± 0.7b	3%	0.0001 ^#^
	Tomato leaves	2.4 ± 2.3a	56%	1.0 ± 1.7b	25%	<0.0001 ^#^
Harmony		7.6 ± 0.5a	----	7.7 ± 0.9a	----	0.9235 ^$^
Global sensations						
Complexity		6.6 ± 0.8a	----	6.5 ± 1.0a	----	0.7432 ^#^
Persistence		7.5 ± 0.8a	----	7.2 ± 1.1	----	0.0108 ^#^

* Different lowercase letters correspond to statistically significant differences at a significance level of 5%. ^#^ *p*-value: *t*-Student with the Welch’s correction for unequal variances according to the *F*-test for sample variance (*p*-value < 0.05). ^$^ *p*-value: *t*-Student test when equal variances could be assumed according to the *F*-test for sample variance (*p*-value > 0.05).

**Table 3:** Mean content (±standard deviation; mg of compound as internal standard equivalents per kg of olive oil) of the chemical classes of volatile compounds detected by gas chromatography (HS-SPME-GC-MS) in olive oils from the two adjacent regions (Côa: 36 oils; and, Douro: 31 oils).

Chemical Family of Volatile Compounds ^§^	Olive Oil’s Geographical Origin (mg/kg)		*p*-Value *
	Côa Valley	Douro Valley	
Alcohols	1.2 ± 3.4b	5.1 ± 4.9a	0.0003 ^#^
Aldehydes	9.8 ± 9.9b	27.7 ± 18.0a	<0.0001 ^#^
Alkanes	1.1 ± 0.7b	2.9 ± 1.7a	<0.0001 ^#^
Alkenes	0.4 ± 0.4b	2.2 ± 0.9a	<0.0001 ^#^
Esters	0.2 ± 0.2b	0.6 ± 0.7a	0.0002 ^#^
Ethers	0.2 ± 0.3b	1.1 ± 0.6a	<0.0001 ^#^
Ketones	0.2 ± 0.2b	0.7 ± 0.7a	0.0004 ^#^
Terpenes	1.4 ± 0.8a	1.4 ± 0.9a	0.9730 ^$^
Total	14.5 ± 11.6b	41.6 ± 19.8a	<0.0001 ^#^

^§^ Peaks identification compounds were identified by comparing the mass spectra fragmentation pattern with the mass spectra database from NIST Standard Reference Database 69, PubChem Compound Summary, and ChemSpider database. Fit and retrofit values were greater than 80%. * Different lowercase letters correspond to statistically significant differences at a significance level of 5%. ^#^ *p*-value: *t*-Student with the Welch’s correction for unequal variances according to the *F*-test for sample variance (*p*-value < 0.05). ^$^ *p*-value: *t*-Student test when equal variances could be assumed according to the *F*-test for sample variance (*p*-value > 0.05).

**Table 4:** Quantification of the contents of aldehydes and alcohols emitted by olive oils originated from Côa Valley (36 oils) or Douro Valley (31 oils), and of the respective total volatiles content (alcohols + aldehydes + alkanes + alkenes + esters + ethers + ketones + terpenes): predictive performance of the MLRMs developed based on sub-sets of feature extracted variables from the electrical resistance signal curves acquired by the nine-MOS sensors comprised on the lab-made E-nose, which were selected using the SA algorithm.

**Geographical Origin**	**Volatiles Chemical Class**	**Concentration Range** **(mg/kg oil) ^a^**	**Nº of Feature Extracted** **Variables ^b^**	**E-Nose-MOS-SA Models (LOO-CV ^c^)**	
				**Determination** **Coefficient (*R*^2^)**	**Root Mean Square Errors** **(RMSE, mg/kg Oil)**
Côa Valley	Alcohols	[0.17, 19.1]	20 ^d^	0.986	0.40
	Aldehydes	[0.41, 64.4]	25 ^e^	0.982	1.34
	Total	[6.24, 78.8]	25 ^f^	0.990	1.19
Douro Valley	Alcohols	[1.11, 22.1]	21 ^g^	0.998	0.23
	Aldehydes	[10.9, 83.9]	20 ^h^	0.984	2.29
	Total	[20.2, 104.9]	19 ^i^	0.981	2.79

^a^ Experimental contents (in mg of compound expresses as internal standard equivalents/kg of oil) determined by HS-SPME-GC-MS, ^b^ Number of feature extracted variables included in the MLRMs, ^c^ LOO-CV: leave-one-out cross-validation quality data (internal validation procedure) for the established MLRMs, ^d^ Feature extracted variables included in the MLRM: S2_LP, S8_LP, S1_INT, S3_INT, S7_INT, S5_MAX, S8_MAX, S9_MAX, S1_MIN, S5_MIN, S2_SUM, S8_SUM, S9_SUM, S2_MEAN, S3_MEAN, S4_MEAN, S5_MEAN, S5_SD, S6_SD, S9_SD, ^e^ Feature extracted variables included in the MLRM: S2_LP, S1_INT, S3_INT, S5_INT, S7_INT, S8_INT, S9_INT, S3_MAX, S4_MAX, S6_MAX, S9_MAX, S1_MIN, S2_MIN, S4_MIN S1_SUM, S4_SUM, S1_MEAN S2_MEAN, S4_MEAN, S5_MEAN, S6_MEAN, S8_MEAN, S6_SD, S8_SD, S9_SD, ^f^ Feature extracted variables included in the MLRM: S3_LP, S4_LP, S6_LP, S8_LP, S9_LP, S1_INT, S3_INT, S6_INT, S8_INT, S2_MAX, S3_MAX, S4_MAX, S6_MAX, S9_MAX, S2_MIN, S8_MIN, S2_SUM, S6_SUM, S7_SUM, S1_MEAN, S2_MEAN, S4_MEAN, S6_MEAN, S5_SD, S6_SD, ^g^ Feature extracted variables included in the MLRM: S1_LP, S4_LP, S5_LP, S6_LP, S7_LP, S4_INT, S5_INT, S6_INT, S7_INT, S2_MAX, S4_MAX, S5_MAX, S6_MAX, S7_MAX, S9_MAX, S1_MIN, S2_MIN, S4_MIN, S7_MIN, S8_MIN, S9_MIN, S2_SUM, S3_SUM, S6_SUM, S5_MEAN, S7_MEAN, S6_SD, S7_SD, S8_SD, ^h^ Feature extracted variables included in the MLRM: S2_LP, S8_LP, S9_LP, S2_INT, S6_INT, S7_INT, S7_MAX, S8_MAX, S3_MIN, S4_MIN, S5_MIN, S7_MIN, S9_MIN, S5_SUM, S9_SUM, S7_MEAN, S6_SD, S7_SD, S8_SD, S9_SD, ^i^ Feature extracted variables included in the MLRM: S5_LP, S7_LP, S3_INT, S6_INT, S7_INT, S8_INT, S4_MAX, S6_MAX, S8_MAX, S4_MIN, S3_SUM, S4_SUM, S8_SUM, S1_MEAN, S4_MEAN, S6_MEAN, S7_MEAN, S9_MEAN, S8_SD.

## Data Availability

Data is contained within the article and additional data can be available upon request.
